# Rice Bran Makes a Healthy and Tasty Traditional Indonesian Goat Meatball, ‘Bakso’

**DOI:** 10.3390/foods10081940

**Published:** 2021-08-20

**Authors:** Rio Olympias Sujarwanta, Michel Mubiayi Beya, Desi Utami, Jamhari Jamhari, Edi Suryanto, Ali Agus, Heather Eunice Smyth, Louwrens Christiaan Hoffman

**Affiliations:** 1 Centre for Nutrition and Food Sciences, Queensland Alliance for Agriculture and Food Innovation (QAAFI), The University of Queensland, Coopers Plains, Brisbane, QLD 4108, Australia; michel.beya@uqconnect.edu.au (M.M.B.); h.smyth@uq.edu.au (H.E.S.); louwrens.hoffman@uq.edu.au (L.C.H.); 2 School of Agriculture and Food Sciences, The University of Queensland, Gatton, QLD 4343, Australia; desi.utami@uq.net.au or; 3 Department of Animal Products Technology, Faculty of Animal Science, Universitas Gadjah Mada, Yogyakarta 55281, Indonesia; jam_hari@ugm.ac.id (J.J.); edi_ugm@ugm.ac.id (E.S.); 4 Department of Agricultural Microbiology, Faculty of Agriculture, Universitas Gadjah Mada, Yogyakarta 55281, Indonesia; 5 Department of Animal Nutrition and Feed Science, Faculty of Animal Science, Universitas Gadjah Mada, Yogyakarta 55281, Indonesia; aliagus@ugm.ac.id; 6 Department of Animal Sciences, University of Stellenbosch, Private Bag X1, Matieland, Stellenbosch 7602, South Africa

**Keywords:** rice bran, tapioca, goat, meatball, starch

## Abstract

Meatballs are popular in Asia and traditionally made from beef or chicken with tapioca (≈8% *wt/wt*) as filler. Tapioca has a high glycaemic index (GI); therefore, rice bran was evaluated as a substitute to create a healthier meatball of acceptable quality. Substitution of tapioca with rice bran (100:0; 75:25, 50:50; 25:75; 0:100% tapioca: % rice bran) decreased the starch content (7.8 to 3.3%) and GI (56.08 to 43.85) whilst increasing the protein (10.9 to 12.8%) and fibre (8.1 to 10.3%) contents. Although consistency (995 to 776 N/mm) was affected, firmness (90.6 to 90.5 N) and shear force (300 to 312 N) were only slightly affected by the ratio of tapioca to rice bran. Sensory analysis revealed that the goat meatball with the substitution of tapioca with up to 25% rice bran was deemed acceptable by 40 Indonesian consumers.

## 1. Introduction

Meatballs are restructured meat products made from ground meat which is typically mixed with starch and some seasonings and formed into round balls (as big as a Ping-Pong ball) and boiled until well cooked [1]. This meatball is very popular among Asian consumers, including Indonesian consumers across all social classes [2]. A search on YouTube on 1 June 2021 with the keyword ‘bakso’, an Indonesian term for meatball, resulted in a hit on numerous videos on this food product; one of these had been watched at least 5.8 million times, indicating how popular this meat product is (https://www.youtube.com/watch?v=BJpGEtyVCII (accessed on 1 June 2021)). The meat products used for ‘bakso’ are either ground beef, buffalo, pork, goat, or poultry meat. The production of ‘bakso’ from goat is not as common as that from beef and chicken, even though, based on Meat and Livestock Australia’s (MLA) latest report, the average goat meal in Indonesia places this country as the third-largest global consumer of goat meat [3]. Goat, as animal food, is recognised to be rich in protein containing little fat and cholesterol [4]. According to USDA [5], lean goat meat consists of about 75.4% moisture, 20.0% protein, 3.1% fat, and 1.1% ash—this indicates that goat has similar protein content to beef and chicken. Despite this fact, goat meat has a lower fat content which is only one-third of that of beef and less than half of chicken meat [5]. While for cholesterol, goat meat has been reported to have the lowest content (75 mg), compared to chicken (90 mg), or beef (86 per 100 g meat of cooked meat) [4]. Goat meat is, therefore, a healthier option, compared to other red meat groups; goat meat has the potential to substitute beef and chicken meat in the production of traditional ‘bakso’.

Typically, ‘bakso’ are made from traditionally farmed species as protein sources, whilst tapioca is used as plant starch. The use of pork as the meat protein source is somewhat limited due to religious beliefs (Halal), and therefore, the use of alternative meat proteins such as goat meat is on the increase. An important ingredient of ‘bakso’ is cassava starch—typically used to produce a chewy sensation. However, the nutritional value of this starch is less desirable due to having a high content of carbohydrate (87%) while being low in protein (1.23%) and fibre (0.2%), as reported by Ijioma [6]. Consumers are becoming increasingly aware of consuming a healthier diet as scientific reports on the relationship between diet and overweight-related diseases (e.g., diabetes) become more prevalent; colorectal cancer is also linked to a diet with too low an intake of fibre [7]. The obesity rate in the world in 2016 was over 650 million people, which could be found both in developing and developed countries [8]. In Indonesia, as a developing country, 21.8% of the adults are classed as being obese [9], while in a developed country such as Australia, 31% of Australian adults were obese in 2017 [10]. Therefore, an exploration of a nutritional meal with lower fat, a lower glycaemic index, and high fibre is desirable to help global people lead a better life.

The glycaemic index (GI) for cassava starch is around 70 [11], while an alternative to this starch is rice bran, with a GI of 19 [12]. Rice bran, a by-product of the milling process, contains 11–15% crude protein, crude fat 15–19%, and crude fibre 7–11% [13]. Indonesia is one of the three largest rice-producing nations globally, producing 56.54 million tons of milled rice in 2018 [14]. This resulted in 5.5 million tons/year of rice bran, a by-product of this milling process [15], which is generally only used for animal feed and not as a food source. It would be of great value if the use of this by-product can be upstreamed through which it becomes a sought-after product fit for human consumption rather than a low-value by-product traditionally used in animal nutrition. Rice bran is the cuticle between the paddy husk and rice grain produced during rice milling and commonly marketed as a cheap animal feed [16]. Rice bran contains high crude fibre, crude protein, zinc, iron, folic acid, and since it is a plant, it has no cholesterol. It is also known to have antioxidant compounds such as polyphenols, phytosterols, and vitamin B and is becoming more popular as a functional food. Even though studies of the nutritional value of rice bran have been conducted such as fermentation and functional rice bran [17], the utilisation of rice bran as a partial replacement of more traditional starch sources in goat meatballs (‘bakso’) is still absent. Rice bran has the potential to be a replacement for cassava starch due to its nutritional value and abundance. The objective of this research was to observe the effect of rice bran on the functional and consumer sensory quality of goat meatball when replacing the traditional starch source (tapioca).

## 2. Materials and Methods

### 2.1. Raw Materials

Goat meat trimmings derived from at least 30 carcasses slaughtered according to halal procedures were sourced from a commercial abattoir, vacuum packed, and stored frozen at −20 °C until used. The proximate composition of the meat, rice bran, and tapioca as the base materials of this research are presented in Table 1.

### 2.2. Meatball Preparation

The basic ingredients (by weight) of the meatballs were ground goat meat (56.3%), ice flakes (22.5%), rice bran and/or tapioca starch (8.1%), 6.7% eggs, 4.0% fried shallot, 1.4% salt, 0.6% sugar, 0.5% fried garlic, 0.4% agar powder, 0.3%, and white pepper. The rice bran and tapioca starch were added in different ratios from 0%, 25%, 50%, 75%, and 100% (Table 2). The five formulations based on the tapioca starch and rice bran compositions each had four replicates/batches weighing 1777g. Each replicate’s ground meat was thawed overnight at 4 °C. A half portion of ground meat was mixed with ground fried shallot and garlic using a Kenwood Major Premier food processor (DeLonghi Australia Pty Limited, NSW, Australia) for 1 min at speed 3. Into this mixture, the other half of the ground meat, salt, pepper, sugar, 2 eggs, agar, tapioca and/or rice bran, and crushed ice-water (400g) were added and mixed thoroughly using the same processor for 3 min at speed 3. From each formulation/treatment and batch/replicate, approximately 100 g raw meatballs were subsampled for chemical analysis. The remaining dough was then handmade (https://www.youtube.com/watch?v=BJpGEtyVCII (accessed on 10 January 2021)) into meatballs with a diameter of approximately 3 cm and a weight ± 20 g. These meatballs were boiled in a water bath at an initial temperature of 100 °C for 5 min until all the meatballs floated and then drained for one minute. During the draining, the internal temperature of five meatballs for each replicate was measured.

### 2.3. Chemical Analyses

After cooking, five to six meatballs per replicate were collected and homogenised prior to chemical analyses. Each raw meatball batter’s replicate was also homogenised further before chemical analyses. Moisture content was analysed following the AOAC (2012) [18] Official Method 934.01 by drying the samples in an oven for 48 h at 105 °C and measuring the weight of the meatball before and after the water was removed. Ash content was observed by combustion of the moisture-free meatball samples at 550 °C in a furnace (Modutemp Pty. Ltd.; Perth, WA, Australia) for 8 h [19].

The neutral detergent fibre (NDF) was determined as follows: an empty filter bag (F57, Ankom Technology, New York, NY, USA) was weighed (W1). Moisture-free cooked meatball samples (0.45–0.50 g) were ground and placed into the weighed bag, and the weight thereof was recorded (W2). Each filter bag was heat-sealed completely (His, Ankom Technology, New York, NY, USA). All bags were placed into a container with 200 mL of acetone, shaken 10 times, and left to stand at room temperature for 10 min. The acetone was discarded and replaced with new acetone, and the process was repeated. After removal of the acetone, the bags were air-dried before being placed on the bag suspender trays that were placed into the Ankom instrument (Ankom2000 with 65 rpm agitation, Ankom Technology, New York, NY, USA). A neutral detergent solution [made from 30 g of sodium dodecyl sulphate, 18.61 g of sodium borate, 4.56 g sodium phosphate dibasic (anhydrous), and 10.0 mL triethylene glycol to which 1 L of distilled water was added (2 L) was added into the digestion vessel, followed by 20 g of sodium sulphite and 4.0 mL of α-amylase and the mixture incubated for 75 min. When the digestion was completed, 2000 mL of boiled water was added to rinse the bags and 4.0 mL of α-amylase was added. All sample bags were transferred into a 250 mL beaker with sufficient acetone and soaked for 5 min, after which the bags were air-dried for 3 h and placed into an oven at 105 °C to dry completely. The filter bags were removed and placed into a desiccator to allow cooling before weighing (W3). The blank bag correction was prepared as a running average of end oven-dried weight divided by the original blank bag weight which counted as C1. The percentage of NDF was calculated according to the following:

$$\frac{100\;\times \;\left(W3-\left(W1\times C1\right)\right)}{W2}$$

The method for analysing the acid detergent fibre (ADF) was similar to that for NDF; however, the reagents were changed. Acid detergent solution (20 g cetyl trimethylammonium bromide (CTAB) to 1 L 1.00 N H2SO4) replaced the neutral detergent solution. Additionally, after completing the process, 4.0 mL of α-amylase was not added during the rinsing process.

The starch contents of the meatball samples were determined as described by [20]. Briefly, 100 mg ground sample was added into a tube to which 0.2 mL of 80% ethanol was mixed and the resulting mixture vortexed. Immediately thereafter, 3.0 mL of α-amylase was added, followed by incubating the mixture for 6 min in a boiling water bath. At 2 and 4 min of incubation, samples were vortexed and returned to the water bath. After 6 min, the samples were once more vortexed (Vortex Genie2, Scientific Industries, Palmerston North, New Zealand) and then transferred to a 50 °C water bath after adding 0.1 mL amyl glucosidase and incubated for 30 min. The samples were then transferred to a 100 mL measuring flask, and the tube was rinsed using distilled water to make up the volume. A 2.0 mL sample was then removed and placed into a 10 mL centrifuge tube and centrifuged for 10 min at 3000 rpm; then, 0.2 mL of the clear suspension was placed into a new plastic tube, wherein 6 mL of glucose oxidase-peroxidase (GOPOD) reagent was added, and this mixture was incubated at 50 °C in a water bath for 20 min; after the absorbance, it was measured at 510 nm (Shimadzu, UV-2550, Manassas, VA, USA).

Protein content was analysed using a LECO CN928 carbon/nitrogen combustion analyser (Leco, St Joseph, MI 49085, MI, USA). A 0.3 g defatted dry sample was combusted at a temperature of 1000 °C. All samples were combusted on the same day. The crude protein content of the sample was estimated by multiplying the N content with a factor of 6.25.

Fat content was analysed using chloroform–methanol, as described by Lee et al. (1996) [21]. A homogenised cooked meatball sample weighing 5.00 g was placed into a 100 mL beaker. Exactly 40 mL chloroform/methanol (2:1) was added to the sample and stirred for one minute. The mixture was filtered through a Whatman #4 filter paper into a separation funnel. An additional 10 mL of chloroform/methanol (2:1) was used to rinse the beaker and filter paper. Into a separation funnel, 20 mL of 5% NaCl was added and mixed thoroughly before allowing the mixture to stand for 30–60 min until the separation line was visible. Exactly 5 mL of the bottom layer was collected into a preweighed beaker, dried in a fume cupboard, and weighed.

### 2.4. Physical Analyses

The Hamm press method was used to measure the water holding capacity of the meatballs. From the homogenised cooked meatballs, 0.3 g from each sample was placed onto the centre of a labelled filter paper disc. The sample was then pressed between Perspex plates (standard pressure: 588 N) using a leverage locking system for exactly 60 s, and a digital photograph of each sample showing seepage liquid and squashed meatball areas were taken. The drip area was calculated using ImageJ software (http://rsbweb.nih.gov/ij/ (accessed on 8 September 2020)) by calculating the outer area minus the inner area. The WHC was calculated using the following equation [22]:

$$\mathrm{WHC}\;:1-\frac{\mathrm{meat}\;\mathrm{area}}{\mathrm{spread}\;\mathrm{of}\;\mathrm{juice}\;\mathrm{area}}$$

Measurements of meatballs’ texture were performed on a computer-assisted texture analyser (Lloyd TA1, Instruments, Bognor Regis, West Sussex, UK) equipped with a 250 N load cell. The settings of the equipment for the first test (measuring consistency and firmness) were as follows: preload at a speed of 21 mm/min; a test speed of 100 mm/min; distance (compression) travelled = 50%; preload stress of 5.6 N. Consistency was defined as the total energy per unit distance (work) used for the 50% compression and firmness as the maximum amount of energy required to distort the meatball at 50% compression. For the second measurement, the shear force required to shear through the whole meatball at a speed of 100 mm/min was determined. Cooked meatball samples were stored overnight in refrigeration condition at <5 °C. Thereafter, the samples were maintained at room temperature for 1 h prior to the textural analysis [23]. Five samples for each treatment’s replicate were evaluated for each of the two assessments performed to compare the meatballs texture analysis: first, a single one-bite was performed to determine consistency and firmness using a compression plate with 100 mm diameter. Second, a shear test was performed using a V-shaped blade to calculate the shear force required to cut through the sample [24].

Colour measurement was carried out as described by Erdem et al. (2020) [25], with modification, using a chromameter (CR-400/4310 Thermo Fisher Scientific Australia Pty Ltd., 5 Caribbean Drive, Scoresby, VIC 3179, Australia) set at d:0° (diffuse illumination/0° viewing angle; specular component included) with a standard observer angle of CIE: 2° and illuminate/observer angle: D-65/10. The chromameter was placed on a white tile to calibrate as per the supplier’s instructions [24]. Cooked samples were stored overnight in refrigeration condition at <5 °C. Five samples for each treatment’s replicate were cross-sectioned, and the colour measurement was taken at the core. The colour reading includes lightness (L), redness (a), and yellowness (b). Average colour values were calculated from five samples per replicate.

Water activity was measured using a water activity metre (Lab Touch-aw, Novasina AG, Lachen, Switzerland) at room temperature [26].

### 2.5. Glycaemic Index Estimation

The estimation of the glycaemic index (GI) value of the ‘bakso’ meatballs used the Formula (a), as described by William et al. [27]. To obtain the GI, the carbohydrate content of each ingredient in the recipe was determined. The carbohydrate values of the ingredients were obtained from a food nutrition database [28]. In order to quantify the amount of carbohydrate each ingredient contributed to the meatball recipe, the total grams of carbohydrate were divided by the gram contributed by that ingredient, and then the proportion for the components was multiplied by the standard GI of the component as obtained from the nutrition database [28]. The GI for agar was obtained from [29]. The calculated GI values from each component were then summed to obtain the total GI of the meatball. The calculation of GI was as follows:

*(a) Food GI = {[GIfood a × g available carbohydrate food a] + [GI food b × g available carbohydrate food b] +* *[GI food c × g available carbohydrate food c] + …}/total g available carbohydrate*

### 2.6. Consumer Sensory Evaluation

This research was approved by the University of Queensland’s Human Research Ethics Committee (HREA ethics approval number 2020001685). To evaluate consumer acceptance of the goat meatball with the addition of rice bran, 40 Australian-based Indonesian participants were recruited locally both in Gatton and St. Lucia, Queensland, who were regular consumers of meatballs, both previously when living in Indonesia and now in Australia. To reach the participants, an Indonesian community WhatsApp group exists (in which two of the coinvestigators participate) that was enabled to communicate with the members. Consumers were aged between 20 and 60, with an average of 35 years old, there were 23 male and 17 female participants. The general demographic profile of the 40 Indonesian consumers is shown in Table 3.

Two sessions were held at The University of Queensland (UQ) each at the St Lucia Campus and Gatton Campus’ using a ‘board room’ style setup; both sessions were from the Indonesian student community, and no participant partook in both sessions. Participants were asked to attend one 1 hr session and tasted no more than 10 meatballs during the session. During sessions, participants were asked to provide informed consent and provided instructions on the session format and consumer questionnaire. Participants were informed to consume as much or as little as they wished of each meatball to complete their assessment. Participants were provided morning tea as an incentive for their participation.

The 40 consumers were divided into groups of 8, with each group receiving a different replicate of each treatment. Participants were asked to score the sample for acceptability of flavour, colour, juiciness, texture, taste, and overall palatability using a 9-point hedonic scale, where 8 is ‘like extremely’, 7 is ‘like very much’, 6 is ‘like moderately’, 5 is ‘like slightly’, 4 is ‘neither like nor dislike’, 3 is ‘dislike slightly’, 2 is ‘dislike moderately’ and, 0 is ‘dislike extremely’. In addition, participants were also provided a detailed demographics questionnaire (age, gender, household income, number or persons in household, and education level) and a line of questions related to protein consumption behaviour.

Prepared meatballs were consumed within three days of preparation. Meatballs were removed from cold storage (4 °C) at least 60 min prior to heating for evaluation. The meatballs were served warm by reheating in a microwave (Homemaker, 900 W) for 60 sec and presented immediately (within 5 min of heating). Individual randomly numbered single meatball samples (approx. 20 g per serving after reheating) were presented to consumers on a plastic plate together with a plastic fork, whilst filtered water for refreshing the palate between samples was also provided. Replicates from all five different sample treatments were presented to consumers according to a randomised design, with at least 2 min between each sample. In total, each replicate from each treatment was evaluated by 20 consumers.

### 2.7. Statistical Analysis

The experiment was carried out with five treatments, each with four replications. The data obtained from chemical analysis (moisture, ash, protein, fat, fibre-NDF, and fibre-ADF), and physical analysis (raw meatball weight, cooked meatball weight, meatball temperature, consistency, firmness, shear force, colour, water activity water holding capacity) were analysed by one-way analysis of variance (ANOVA) in Statistica^®^ 13.5.0.17 (TIBCO™ Software inc. http://tibco.com (accessed on 26 October 2020)). For post hoc analysis, Fisher’s least significant difference (LSD) test was utilised. For the sensory analyses (overall, flavour, colour, juiciness, texture, taste, and odour), a mixed model ANOVA with panellist and session as random effects, and treatment as a fixed effect, were conducted on the R package (lmerTest’s version 3.1-3; https://github.com/runehaubo/lmerTestR (accessed on 26 October 2020)). Main effects and interactions with a *p* ≤ 0.05 are reported as significant. Principal component analysis (PCA) was performed to study the relationship between chemical characteristics and physical properties.

## 3. Results

**Figure 1:**
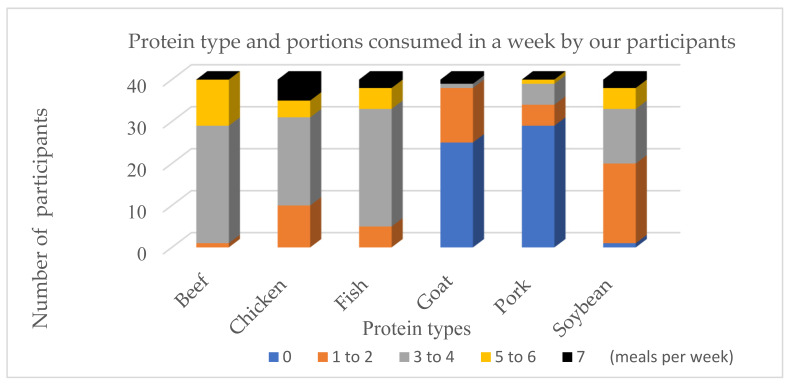
Protein source types and frequency of its consumption by 40 participants. Protein sources including beef, chicken, fish, goat, pork, and soybean, and frequency meals per week including 0, 1–2, 3–4, 5–6, and 7 times per week.

**Figure 2:**
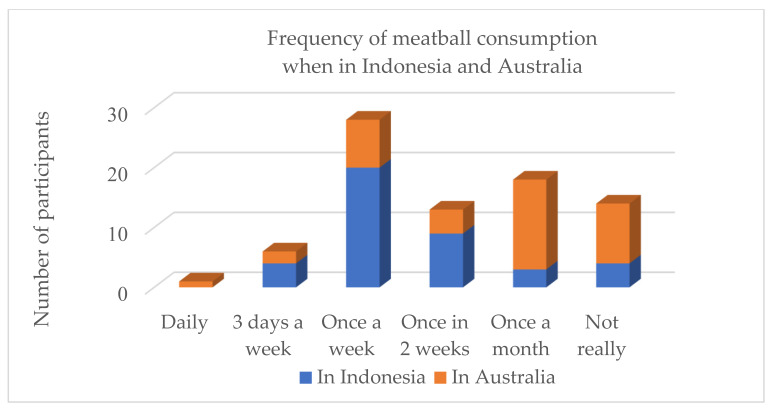
Frequency of meatball consumption both when stay in Indonesia and Australia of our participants, i.e., 40 Indonesians who currently reside in Australia.

The proximate composition of the basic ingredients used to make the goat meatballs and the general demographic profile of the Indonesian consumers are shown in Table 1 and Table 3, whilst Figure 1 and Figure 2 report the protein consumption profile and meatball consumption frequency of our participants, respectively. The statistical analysis results of the chemical analysis, physical analysis, and sensory analysis for meatball substituted by various levels of rice bran are presented in Table 4. Goat meat contained up to 20.9% protein with a fat content of 11.8%, while the rice bran had 13.6% protein, 16.9% fat content, and fibre-NDF of 28.8%, and fibre-ADF of 17.4%. Tapioca had no protein and fat, whilst both fibre-NDF and ADF were <4.0%.

The weight of raw meatballs did not differ between the five treatments; however, after being boiled, the weights differed (*p* = 0.05; Table 4). When tapioca was fully replaced (100%) with rice bran, the average boiled meatball weights were the lightest. The temperatures of the different meatball treatments after boiling did not differ (*p* = 0.72). The moisture contents of the raw dough did not differ between the treatments. In contrast, the moisture in the cooked meatball decreased as the level of rice bran increased (*p* = 0.01). Treatment influenced the protein contents of both the raw and cooked meatballs. Although the protein content varied between the ratio of tapioca and rice bran (*p* = 0.02), the two treatments with the higher levels of tapioca had lower protein contents that did not differ from each other (11.6–11.9%) than the higher-level rice-bran-containing treatments (13-5-14.4%), the latter not differing from each other. It was reported that adding rice bran to the fermented cereal-based food product also increased the protein content significantly [30].

Fat content ranged from 8 to 11% in the cooked meatballs; however, it did not differ both in the raw (*p* = 0.67) and cooked (*p* = 0.15) meatballs. Ash contents in both raw and cooked meatballs increased with decreasing levels of tapioca. The substitution of rice bran decreased the amount of starch considerably (*p* < 0.01), which ranged from 7.8% in the 0% rice bran treatment to 3.3% in 100% rice bran meatballs. The fibre-NDF and -ADF also varied among the treatments (*p* = 0.10), with a slight rise with the addition of rice bran from 35.7% to 38.3% for fibre-NDF and 8.1%4 to 10.4% for fibre-ADF.

The calculation of GI values resulting from the replacement of tapioca starch by rice was 56.08 for 0%, 53.49 for 25%, 50.62 for 50%, 47.42, for 75%, and 43.85 for 100% rice replacement, respectively. As indicated in Table 4, 100% of rice bran substitution showed the highest water holding capacity (WHC) (*p* <0.01); as the amount of rice bran added decreased, the WHC also decreased. The water activity also decreased with an increase in rice bran (*p* = 0.01); this would indicate that the products with high bran % should be more microbial shelf stable. The texture analysis results are presented in Table 4. The consistency (*p* = 0.01) and firmness (*p* = 0.01) parameters differed between the treatments. The consistency (N/mm) decreased as the rice bran substitution increased; however, the firmness (N) indicated inconsistent results, as the treatment of 50% rice bran showed the highest firmness, while the lowest firmness was found in the 100% substitution by rice bran. The shear force (N) (*p* > 0.05) did not differ between treatments.

The L* (lightness) values of the treatment without rice bran replacement was the lowest (*p* = 0.11), compared to other treatments; however, there was no fixed trend noticeable. The effect of rice bran substitution on a* (redness) values of the goat meatball differed (*p* = 0.04), although the values’ differences were numerically minimal. The b* (blue to yellow) values differed among the treatments (*p* < 0.01), increasing as the rice bran substitution increased. The substitution by rice bran showed varying effects on the sensory properties as indicated by the Indonesian consumers. The consumer acceptance scores of colour (*p* = 0.21) and odour (*p* = 0.15) did not differ between the treatments of various ratios of starch to rice bran. The flavour (*p* = 0.03), juiciness (*p* < 0.01) and texture attributes showed similar results; the ratio of rice bran substitution from 75% to 100% were rated lower. Juiciness showed unexpected results (*p* < 0.01), where consumers gave a higher score on the 50% of rice bran substitution rather than 0% and 25%. The texture attribute decreased (*p* < 0.01) as the amount of rice bran substitution increased. The ‘overall’ attribute differed amongst the treatments (*p* < 0.01); however, the substitution of rice bran up to 50% did not affect the ‘overall’ consumer acceptance scores, whereas a higher % substitution led to a decreased score.

Protein consumption profiles for each Indonesian participant is displayed in Figure 1, with six categories of protein source—beef, chicken, fish, goat, pork, and soybean product. Beef product was the most widely consumed protein source, followed by chicken, while pork was rarely consumed. More than 70% of participants consumed beef more than three times a week; in contrast, more than 70% of the participants surveyed have never eaten pork as their protein source. Lamb or goat meat consumption was also very low—62.5% of consumers stated that they rarely consumed this protein source.

Meatball consumption behaviour, both in Indonesia and Australia, was also part of the consumer questionnaire. Most participants (60%) consumed meatballs at least once a week while in Indonesia, and more than 25% of them consumed meatballs in Australia once a week, indicating that meatballs are very popular among the participants (Figure 2).

**Figure 3:**
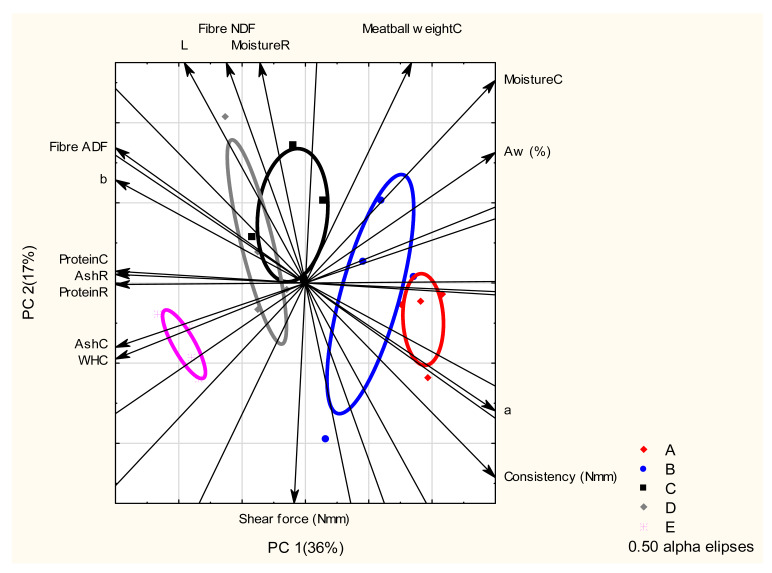
Principal component analysis (PCA) biplot indicating the means of the associations between chemical characteristics and physical properties of five treatments of tapioca substituted with rice bran: A = starch: rice bran (100:0); B = starch: rice bran (75:25); C = starch: rice bran (50:50); D = starch: rice bran (25:75); E = starch: rice bran (100:0).

Principal component analysis (PCA) biplot demonstrates the means of the relationship between chemical properties and physical characteristics of goat meatball substituted by the five rice bran treatments (A: 0% rice bran; B: 25% rice bran; C: 50% rice bran; D: 75% rice bran and E: 100% rice bran) (Figure 3) and indicated that the control treatment (without substitution of rice bran) showed a stronger association with the consistency and a* colour ordinate than the other treatments. The B treatment showed a stronger relationship with water activity and moisture of cooked meatballs. The C treatment with 50% starch and rice bran indicated stronger relationships with fibre-NDF, fibre-ADF, protein content, and moisture content. The 75% replacement of tapioca with rice bran (D treatment) had a close relationship with L*, fibre-NDF, fibre-ADF, protein content, and ash content. Surprisingly, treatment with 100% substitution of rice bran showed stronger relationships between protein content, fibre-NDF, fibre-ADF, WHC, ash content, and shear force.

## 4. Discussion

### 4.1. Chemical Analysis

Typically, the fat content of goat meat ranges between 1.13 and 2.39% (supplemented with grass hay and sunflower cake) [31]; generally, goat meat is lean with lower levels of subcutaneous fat [32]. The goat meat fat in this experiment was high (11.8%; Table 1) as trimming cuts that contained a reasonable amount of visible fat were used; therefore, it was decided to make the goat meatballs without any additional fat added.

There were no major differences among the goat meatball samples in terms of temperature, raw meatball weight, fat in both raw and cooked samples, moisture in cooked meatball, and fibre-NDF and -ADF (Table 4). The moisture content in the boiled/cooked meatball decreased as the replacement of rice bran increased. However, this loss in moisture would most probably be negated when the meatballs are consumed with soy and chili sauces—a typical way of eating ‘bakso’ as a meal in Asia. The protein content in the cooked meatball increased with the addition of rice bran; this increase is directly linked to the protein content of the rice bran, whilst the tapioca had no measurable protein (Table 1). In contrast, the addition of 1–4% of dietary fibre from rice bran decreased the protein content of emulsion-type sausages [33]. During the boiling/cooking process, the meatballs lost ~2% fat, a loss linked to the fat melting/leaching out during the cooking process. It was noted that an increase in total cooking loss was linked to a decrease in goat meat’s fat content [34]

As expected, the addition of rice bran increased the fibre content of the meatballs (Table 4); the ADF-fibre content was 27% higher in the meatball with 100% rice bran and 17% higher in fibre-NDF in tapioca:rice bran (with the ratio of 25:75%) than the 100% tapioca treatment. However, the total contribution of the tapioca and/or rice bran to the recipe was only 8.1%; thus, the reason for the slight change in both ADF and NDF % is depicted in Table 4. In contrast, the starch content decreased with the increase in the percentage of rice bran addition (>50%, compared to the control treatment). Generally, the addition of dietary fibre to meatballs aims to improve the nutritional value by enhancing satiety and reducing energy intake and, at the same time, increase the fibre consumption among consumers; starch typically contributes about 70–80% of the total carbohydrates in normal diets—a value deemed to be too high and thus unhealthy [35]. In chevon patties, for example, it was noted that 15–20% addition of oat bran enhanced the nutritional value with minimal composition and texture changes [36].

### 4.2. Glycaemic Index (GI)

The GI is a ranking of carbohydrates in foods from 0 to 100 depending on how fast and how much the specific carbohydrates increase blood sugar after being ingested [27]. Carbohydrate-based foods are categorised into three groups in which low-GI foods have a GI < 55, and medium-GI food have a GI ranging between 55 and 70, while high-GI food products have a GI of more than 70 [37]. The calculation of GI values resulting from the replacement of tapioca starch by rice bran caused a decrease in the GI from 56.08 for 0% rice bran (100% tapioca), down to 43.85 for 100% rice bran replacement. The treatment with 0% rice bran, which is also the traditional recipe for using tapioca in ‘bakso’, falls in the category of medium-GI foods, whereas all other treatments with rice bran are in the category of food with low GI. These results demonstrated that the inclusion of rice bran in the meatball recipe decreased the overall GI of the ‘bakso’ meatballs. This decrease in the GI of meatballs could be related to the high concentration of fibre in the rice bran. In agreement with these findings, it was demonstrated that the inclusion of dietary fibre in bread reduced its glycaemic index [38]. Moreover, Cheng et al. [39] suggested that rice bran might serve as a useful nutrient to ameliorate the glycaemic anomalies in type 2 diabetes.

### 4.3. Physical Analysis

Texture is an important factor dictating the sensory attributes in meatball products as well as palatability. The effects of various ratios of starch to rice bran on the textural properties of ‘bakso’ are shown in Table 4. Consistency and firmness were (*p* < 0.05) affected by the ratio of starch to rice bran. Consistency had the highest score at 0% and lowest at 50%, and the firmness had the highest score at 50% and lowest at 100% of rice bran in the ratio starch to rice bran. Shear force was not (*p* > 0.05) affected by the increased proportion of rice bran. The change in the textural profile of meatballs with rice bran, compared to the control, could be due to migration and loss of water. A similar observation was made by Ham et al. [40], who found changes in the textural profile of meatballs with the increase of rice bran; however, there was no significant difference in terms of cohesiveness. Additionally, the increase in fibre from the rice bran could have resulted in a decrease in cohesiveness. Adding bael (*Aegle marmelos* L.) pulp residue to goat meat nuggets (emulsions) was noted to decrease the hardiness, gumminess, and chewiness of the products [41]. Similarly, when full-fat soy pastes or textured soy granules were added to goat meat nuggets (the final product was an emulsion), replacing 15% of the goat meat, a decrease in the textural profile (shear force, hardness, springiness, gumminess, and chewiness) was observed—the decrease was contributed to both the soft texture of the soy paste and the increase in water content [42]. A similar decrease in texture attributes was noted by the same research team when soy proteins in the form of full-fat soy paste were added to goat meat patties [43]. The effect of the addition of soy proteins (as well as other additives such as liquid whole egg and fat) into goat patties was modelled, and it was shown that the acceptability of the baked patties decreased as the amount of soy increased [44]—a finding somewhat similar to that from this study with the addition of rice bran.

As pertaining to the colour of the cooked meatballs (Table 4), the yellowness increased with the addition of rice bran, whereas the redness decreased. These results are similar to other research, e.g., in meat products, the yellowness increased with the addition of fibre [23], and the redness was reported to decrease with the increased level of rice bran [45]. Furthermore, Kim et al. [45] demonstrated that the addition of 3% of rice bran in ground pork enhanced the yellowness values. Conversely, Ayhan [46] reported that the addition of cereal bran in meatballs had much lower yellowness than the control samples. Huang et al. [47], who studied the impacts of rice bran in meatballs, showed that the white index decreased with the addition of rice bran, in the present study there was no difference in this colour ordinate (L*) between treatments (Table 4). On the other hand, Das et al. [41] showed that with the addition of bael, the reflectance (lightness) and redness of the goat nuggets increased, whilst the yellowness remained the same. However, when full-fat soya paste was added to goat patties, there was an increase in the yellowness and a decrease in the redness of the final raw product [48].

Adding lower-valued, fibre-rich plant by-products has been known to improve the functional properties of goat meat patties/products [49,50,51]; for example, psyllium husks were shown to improve the functional quality of goat burger patties [52,53].

### 4.4. Consumers Sensory Evaluation

Indonesian people (who had been living in Australia for more than 6 months) were targeted for this study because they are the typical target market for this meatball product in Indonesia. These consumers evaluated the new recipe of ‘bakso’, using goat meat instead of beef as the common ingredient and the additional rice bran to replace the tapioca as filler. The compositional results indicated that rice bran replacement of tapioca resulted in a ‘bakso’ that was healthier (higher protein and fibre contents and lower GI); however, the next question was ‘Would people still be interested to eat such a bakso?’ Consumer acceptability scores for parameters such as colour and odour were not affected by the substitution of the rice bran even up to 100% replacement. It was stated that substitution of cassava starches up to 100% with rice bran in beef meatballs had a significant effect on consumer acceptance in terms of colour attributes [2]. However, in this investigation, the ‘overall’, flavour, texture, and taste parameters did not differ when 0% or 25% of rice bran replaced tapioca, indicating that the latter was still acceptable to the consumers with most of the parameters lying within the ‘like slightly’ to ‘like moderately’ classification. It would seem as if a 25% rice bran replacement of tapioca is considered as optimum for use in a goat meatball. However, traditionally, ‘bakso’ is eaten with various sauces, and it is argued that if the meatballs had been served with different sauces, the different treatments’ ‘bakso’ may have fallen into the higher classification of ‘like very much’ or even ‘like extremely’.

## 5. Conclusions

Substitution of tapioca with rice bran in the production of a traditional Asian goat meatball, ‘bakso’, improved its nutritional quality with an increase in the protein and fibre contents, whilst the starch content was decreased. More importantly, the replacement of tapioca with rice bran resulted in a ‘bakso’ with a low GI, from a medium GI. The goat ‘bakso’ with rice bran was found to be acceptable to the Indonesian consumers, who used the score of ‘like slightly’ to ‘like moderately’ for all the attributes. Up to 25% substitution with rice bran was revealed to be acceptable by 40 Indonesian consumers. It is argued that if the rice bran ‘bakso’ were to be consumed in the traditional manner with sauces, this liking would move up the scale, and the consumers may not be able to distinguish between the different treatments; this warrants further research. Another aspect worthy of investigation is the cost implication of using rice bran, which is presently used as animal feed, in a food source such as ‘bakso’.

As mentioned by Teixeira et al. [45], numerous goat meat products such as ‘bakso’ are part of the culture of various countries and have not yet been studied and characterised for the preservation of their recipes and origins; this paper is a start in describing the very popular and readily consumed (in Indonesia) ‘bakso’.

## Figures and Tables

**Table 1:** Analysed proximate composition (%) of goat meat, rice bran, and tapioca used to make goat meatballs (‘bakso’).

Sample	Moisture	Ash	Protein	Fat	Fibre-NDF	Fibre-ADF
Meat	63.8 ± 0.68	0.9 ± 0.02	20.9 ± 0.48	11.8 ± 1.50	ND *	ND *
Rice Bran	7.5 ± 0.00	9.5 ± 0.00	13.6 ± 0.00	16.9 ± 0.00	28.8 ± 1.05	17.4 ± 1.52
Tapioca	10.8 ± 0.00	0.2 ± 0.00	0.00 ± 0.00	0.01 ± 0.00	3.7 ± 1.15	3.7 ± 0.24

* ND: not determined; NDF: neutral detergent fibre; ADF: acid detergent fibre.

**Table 2:** Recipe for goat ‘bakso’ meatballs with different ratios of tapioca starch to rice bran.

Ingredients (g)	Starch: Rice Bran				
	100:0	75:25	50:50	25:75	0:100
Ground goat meat	1000	1000	1000	1000	1000
Tapioca starch	144	108	72	36	0
Rice bran	0	36	72	108	144
Salt	25	25	25	25	25
Sugar	10	10	10	10	10
Fried garlic	10	10	10	10	10
Fried shallot	55	55	55	55	55
White pepper	6	6	6	6	6
Agar powder	7	7	7	7	7
Ice flakes	400	400	400	400	400
Egg	120	120	120	120	120
Total	1777	1777	1777	1777	1777

**Table 3:** General demographic profiles of the Indonesian consumers evaluating goat ‘bakso’ meatballs made with varying levels of rice bran substituted for tapioca.

Gender	%	Age	%	Household Income (AUD/month)	%	Number of People in Household	%	Education Level	%
Male	57.5	20 or younger	2.5	Below 2000	22.5	1	10	Bachelor	30
Female	42.5	21–30	37.5	2001–3000	47.5	2	20	Master	30
		31–40	52.5	3001–4000	25	3	20	Doctoral	40
		41–50	2.5	4001–5000	5	4	45	Post-doctoral	0
		51–60	2.5	5001–10,000		5	2.5		
		60 years or older	2.5	10,000 or more		6 or more	2.5		
Total (%)	100		100		100		100		100

**Table 4:** Mean (±s.e.) chemical, physical, and sensory values for goat meatballs made with varying ratios of tapioca and rice bran.

Parameter	Tapioca to Rice Bran Ratio					*p*-Value
	100:0	75:25	50:50	25:75	0:100	
**Chemical Analysis**						
Weight (raw) g	1338.4 ± 9.74	1268.1 ± 84.32	1338.5 ± 11.68	13481.3 ± 19.31	1329.2 ± 15.08	
Weight (cooked) g	1393.1 ^a^ ± 15.76	1291.4 ^ab^ ± 86.55	1346.6 ^a^ ± 81.71	1325.3 ^ab^ ± 18.71	1220.0 ^b^ ± 14.66	0.99
Temperature of meatball °C	78.1 ± 1.78	78.7 ± 0.98	76.5 ± 3.61	78.3 ± 0.73	81.3 ± 1.84	0.05
Moisture (raw) (%)	64.0 ± 0.56	64.1 ± 0.48	65.2 ± 0.46	65.2 ± 0.74	64.3 ± 0.10	0.72
Moisture (cooked) (%)	66.9 ^a^ ± 0.48	65.2 ^b^ ± 0.71	65.1 ^b^ ± 0.68	64.9 ^b^ ± 0.51	63.2 ^c^ ± 0.09	0.35
Protein (raw) (%)	10.9 ^b^ ± 0.11	11.2 ^b^ ± 0.59	11.8 ^ab^ ± 0.58	11.7 ^ab^ ± 0.23	12.8 ^a^ ± 0.05	<0.010
Protein (cooked) (%)	11.9 ^b^ ± 0.62	11.6 ^b^ ± 0.24	13.5 ^a^ ± 0.44	13.5 ^a^ ± 0.41	14.4 ^a^ ± 0.43	0.07
Fat (raw) (%)	10.5 ± 0.77	12.5 ± 1.11	11.0 ± 1.47	12.5 ± 0.77	11.1 ± 0.87	0.02
Fat (cooked) (%)	8.7 ± 0.46	10.1 ± 0.56	10.1 ± 0.56	10.2 ± 0.72	10.9 ± 0.75	0.67
Fibre-NDF (DM %)	35.7 ± 0.64	37.6 ± 1.59	37.3 ± 1.73	41.2 ± 1.54	38.3 ± 0.75	0.15
Fibre-ADF (DM %)	8.1 ± 0.64	8.6 ± 0.29	9.2 ± 0.86	10.4 ± 0.96	10.3 ± 0.75	0.1
Starch (cooked)	7.8 ^a^ ± 1.31	7.8 ^a^ ± 0.11	6.2 ^ab^ ± 0.06	5.0 ^bc^ ± 0.23	3.3 ^c^ ± 0.08	0.1
Ash (raw) (%)	2.0 ^d^ ± 0.04	2.1 ^c^ ± 0.03	2.3 ^b^ ± 0.05	2.4 ^b^ ± 0.05	2.6 ^a^ ± 0.05	0.01
Ash (cooked) (%)	1.6 ^d^ ± 0.04	1.7 ^d^ ± 0.05	1.9 ^c^ ± 0.03	2.0 ^b^ ± 0.00	2.3 ^a^ ± 0.03	<0.010
**Physical analysis**						
Consistency (N/mm)	99.5 ^a^ ± 3.59	87.7 ^b^ ± 3.76	71.6 ^c^ ± 5.20	75.7 ^c^ ± 3.52	77.6 ^bc^ ± 2.68	0.01
Firmness (N)	9.0 ^b^ ± 0.02	9.1 ^a^ ± 0.02	9.2 ^a^ ± 0.03	9.1 ^b^ ± 0.05	9.1 ^b^ ± 0.00	0.01
Shear Force (N)	30.0 ^ab^ ± 1.62	27.8 ^abc^ ± 1.22	26.4 ^bc^ ± 1.56	25.8 ^c^ ± 1.34	31.2 ^a^ ± 1.06	0.06
L*	47.0 ^c^ ± 0.77	47.4 ^bc^ ± 0.31	49.2 ^a^ ± 0.41	49.0 ^ab^ ± 0.85	48.7 ^abc^ ± 0.34	0.11
a*	1.6 ^a ±^ 0.15	1.2 ^ab^ ± 0.22	0.8 ^b^ ± 0.21	0.7 ^b^ ± 0.11	0.8 ^b^ ± 0.23	0.04
b*	11.5 ^b^ ± 0.26	11.8 ^b^ ± 0.26	13.7 ^a^ ± 0.24	13.4 ^a^ ± 0.53	14.1 ^a^ ± 0.18	<0.010
Aw	0.984 ^a^ ± 0.0015	0.983 ^ab^ ± 0.0015	0.982 ^ab^ ± 0.0015	0.979 ^bc^ ± 0.0015	0.975 ^c^ ± 0.0015	0.01
Water Holding Capacity	0.02 ^d^ ± 0.020	0.08 ^cd^ ± 0.020	0.13 ^bc^ ± 0.020	0.16 ^b^ ± 0.0120	0.24 ^a^ ± 0.020	<0.010
**Sensory analysis**						
Overall	6.5 ^a^ ± 0.15	6.4 ^a^ ± 0.15	6.1 ^ab^ ± 0.14	5.8 ^b^ ± 0.19	5.7 ^b^ ± 0.20	<0.010
Flavour	6.3 ^a^ ± 0.19	6.2 ^a^ ± 0.18	5.9 ^ab^ ± 0.15	5.7 ^b^ ± 0.18	5.7 ^b^ ± 0.19	0.03
Colour	6.0 ± 0.20	6.3 ± 0.18	6.2 ± 0.17	6.0 ± 0.19	5.8 ± 0.19	0.21
Juiciness	5.8 ^a^ ± 0.15	5.9 ^ab^ ± 0.16	6.1 ^a^ ± 0.17	5.4 ^bc^ ± 0.20	5.2 ^c^ ± 0.27	<0.010
Texture	6.1 ^a^ ± 1.16	5.7 ^ab^ ± 0.23	5.4 ^bc^ ± 0.20	5.5 ^bc^ ± 0.23	5.2 ^c^ ± 0.25	<0.010
Taste	6.2 ^a^ ± 0.20	6.2 ^a^ ± 0.14	6.0 ^ab^ ± 0.18	5.8 ^ab^ ± 0.22	5.5 ^b^ ± 0.20	0.02
Odour	6.1 ± 0.22	6.1 ± 0.16	5.9 ± 0.18	6.0 ± 0.20	5.6 ± 0.15	0.15

All values are expresses as mean ± standard error for chemical, physical analysis, and sensory analysis. ^a,b,c,d^ Means with different superscript letters in the same row differ significantly (*p* ≤ 0.05). NDF: neutral detergent fibre, ADF: acid detergent fibre, DM: dry matter.

## Data Availability

Data available from corresponding author.
